# The effect of deuterated PLK1 inhibitor on its safety and efficacy *in vivo*

**DOI:** 10.3389/fonc.2025.1510052

**Published:** 2025-03-21

**Authors:** Tingyan Zhang, Xiaobing Deng, Haoping Jin, Zhengshu Peng, Yi-Ching Hsueh, Chunming Zhang, Gang Niu, Jianfei Yang

**Affiliations:** ^1^ Phil Rivers Technology, Beijing, China; ^2^ Phil Rivers Biotechnology, Shenzhen Virtual University, Shenzhen, China; ^3^ College of Chemistry and Molecular Engineering, Peking University, Beijing, China

**Keywords:** small molecule inhibitor, deuterated compound, PLK1, PK/PD, safety, efficacy, cancer

## Abstract

**Background:**

The FDA’s approval of deutetrabenazine, the first deuterium-labeled drug, demonstrated an improved safety profile compared to its non-deuterated counterpart, tetrabenazine. While Polo-like kinase 1 (PLK1) inhibitors have shown promise in cancer treatment, current inhibitors face challenges with toxicity and narrow therapeutic windows, highlighting the need for more effective and safer PLK inhibitors.

**Methods:**

The molecule of PR00012 was generated by replacing all the hydrogen atoms with deuterium on piperazine of the molecule NMS-P937. Several critical *in vitro* assays comparing PR00012 and NMS-937 were conducted, including kinase and cellular inhibition, *in vitro* metabolic stability, and permeability. *In vivo*, both compounds were compared for their pharmacokinetics and pharmacodynamics, toxicity and efficacy.

**Results:**

Both compounds exhibited similar characteristics *in vitro*, including the inhibition of six pancreatic cancer cell lines and 416 kinases. PR00012 demonstrated a slightly longer half-life than NMS-P937 *in vivo*. In tumor-bearing mice, PR00012 more significantly reduced phosphorylated TCTP levels in tumors compared to NMS-P937. Importantly, animals treated with PR00012 showed lower toxicity than those treated with NMS-P937. In mice, fewer animals died from PR00012 treatment compared to NMS-P937 treatment across M-NSG, BALB/c nude, and NOD SCID strains. In a 14-day repeated administration toxicity study in Sprague-Dawley rats, one-third of rats died when treated with NMS-P937, while no rats died from PR00012 treatment. In several cell-derived xenograft (CDX) models, PR00012-treated groups consistently showed slightly better tumor growth inhibition in M-NSG, BALB/c nude, and NOD SCID mice.

**Conclusion:**

The deuterated PR00012 demonstrated an improved safety profile and slightly enhanced efficacy compared to its non-deuterated counterpart, NMS-P937. This study provides a foundation for further clinical trials investigating the treatment of various cancers.

## 1 Introduction

An innovative approach to enhancing drugs’ efficacy and safety profile involves the use of deuterium, a stable hydrogen isotope. Deuterium substitution, which is the process of replacing some of the hydrogen atoms in a drug molecule with deuterium atoms, can significantly influence their pharmacokinetic properties due to the deuterium kinetic isotopic effect (DKIE) (1). This effect arises because deuterium, compared to protium (regular hydrogen), has a smaller molar volume and forms shorter carbon-deuterium (C-D) bonds, which are more stable in oxidative processes (2). Such stability can slow down drug metabolism, particularly cytochrome P450 (CYP450)-mediated transformations, enhancing the drug’s bioavailability and reducing toxicity (3).

The clinical potential of deuterated drugs was first realized with the FDA approval of deutetrabenazine (Austedo), a deuterated version of tetrabenazine, in 2017 (4). Compared with tetrabenazine, deutetrabenazine was associated with a significantly lower risk of moderate to severe adverse effects (AEs) (5). Deutetrabenazine’s approval demonstrated that deuterium substitution could improve therapeutic profiles, offering benefits such as longer duration of action and reduced side effects (6). Following Austedo, numerous drug candidates incorporating deuterium have begun development. These deuterated compounds showed potential for improved pharmacokinetic profiles and better therapeutic effectiveness when compared to their traditional, non-deuterated versions (7). Deuterium substitution is particularly beneficial in drugs metabolized quickly, as it can extend their half-life and reduce dosing frequency (7). The potential of deuterium substitution to reduce the formation of toxic metabolites and decrease the potential for adverse effects is a reason for optimism in the pharmaceutical industry. This versatility and potential have sparked a growing interest in deuterium chemistry, with numerous deuterated compounds currently undergoing clinical trials (8).

Polo-like kinase 1 (PLK1) is a serine/threonine-protein kinase that plays a pivotal role in cell cycle regulation, particularly during mitosis (9, 10). Its function is critical for the proper progression of cell division, and aberrations in its activity have been linked to oncogenesis (11). Extensive research has demonstrated that PLK1 is overexpressed in various human cancers, with elevated levels being associated with poor prognosis in tumors such as breast, lung, and colorectal cancers (12). This has positioned PLK1 as a highly promising therapeutic target for the treatment of a variety of cancers (13).

Among the most advanced PLK1 inhibitors in clinical development is NMS-P937, currently named as Onvansertib (14, 15). Onvansertib targets PLK1 while minimizing collateral effects on closely related kinases PLK2 and PLK3; the IC50 of kinase inhibition of PLK2 and PLK3 was more than 5,000-fold higher than that of PLK1 (15, 16). This high level of selectivity is crucial in developing effective cancer treatments with fewer adverse effects. A phase 1 clinical trial demonstrated that NMS-P937 is well tolerated at a given dosage in 5-day dosing time period (17). Onvansertib combined with FOLIFRI/bevacizumab exhibited manageable safety and promising efficacy in second-line treatment of patients with KRAS-mutant metastatic colorectal cancer (18). In another phase 1b study, the onvansertib and decitabine combination was well tolerated and demonstrated antileukemic activity, particularly in patients with target engagement and decreased mutant ctDNA following treatment (19). However, all of these trials have used a dosing regimen of 5-day dosing and 9-day resting (18, 19), indicating the tolerability is relatively low. Thus, further PLK1 inhibitor modification is needed to improve the tolerability and efficacy.

In this study, we describe a deuterated PLK1inhibitor, PR00012, based on the compound Onvansertib. We discuss the characterization of the deuterated compound, its pharmacokinetic advantages, the *in vivo* toxicity and efficacy, as well as the potential impact on clinical outcomes.

## 2 Materials and methods

### 2.1 Compounds

Both NMS-P937 and the deuterated version, PR00012, were synthesized at ChemPartner (Pudong, Shanghai, China). For cell culture and kinase assays, the compounds were dissolved in DMSO and diluted at a 3-fold or 5-fold gradient starting from a concentration of 10 μM, to achieve the final concentrations for the assays.

### 2.2 Cell culture

Human pancreatic cancer cell lines (PSN1, PANC-1, Panc 04.03, MIA PaCa-2, BxPC-3, and Capan-1) were cultured in their respective culture media. PSN1 cell lines were maintained in RPMI 1640 + 10%FBS. MIA PaCa-2 cells were cultured in DMEM+10%FBS+5% horse serum. Panc 04.03 cells were grown in RPMI 1640 + 15%FBS+10μg/mL Insulin. PANC-1 cells were maintained in DMEM+10%FBS. BxPC-3 cells were cultured in RPMI 1640 + 10%FBS. Capan-1 cells were grown in IMDM+20%FBS. All cells were incubated at 37°C in a humidified atmosphere containing 5% CO_2_.

For assays, cells in the logarithmic growth phase were harvested, seeded to different concentrations at 2,000-6,000 cells/well in 96-well plates. Following overnight incubation, test compounds were added at various concentrations and incubated for 72 hours. Cell viability was assessed by the ATP concentration using the CellTiter-Glo reagent (Promega, Madison, WI 53711 USA), and luminescence values were recorded by a Multilabel Reader (PerkinElmer, Waltham, MA, USA).

### 2.3 Kinase assays

Test compounds PR000012 and NMS-P937 were dissolved in DMSO and thoroughly mixed by vortexing. Serial dilutions in DMSO were prepared. PLK1 was serially diluted 5-fold from 10 μM, yielding concentrations from 10000 nM to 0.00512 nM. Other targets were serially diluted 3-fold from 10 μM, yielding concentrations from 10000 nM to 0.5 nM. We have used two kinase assays: HTRF assay and ADG-Glo assay.

For HTRF Assay, 2× ATP/substrate solution and 2× kinase solution were prepared in kinase reaction buffer. Using an Echo 655, 50 nL of each diluted compound was transferred to a 384-well detection plate. The plate was centrifuged at 1000 rpm for 1 minute. 2.5 μL of 2× kinase solution was added to each well. The plate was incubated at 25°C for 10 minutes. 2.5 μL of 2× ATP/substrate solution was added to each well. The plate was centrifuged at 1000 rpm for 1 minute. The plate was incubated at 25°C for 40 minutes. 2× XL665 and antibody detection reagent were prepared in detection buffer. 5 μL of the prepared kinase detection reagent was added to each well. The plate was centrifuged at 1000 rpm for 1 minute. The plate was incubated at 25°C for 1 hour. Fluorescence signals at 620 nm (Cryptate) and 665 nm (XL665) were measured using a multifunction plate reader.

For ADP-Glo Assay, 2× ATP/substrate solution and 2× kinase solution were prepared in kinase reaction buffer. Using an Echo 655, 40 nL of each diluted compound was transferred to a 384-well detection plate. The plate was centrifuged at 1000 rpm for 1 minute. 2 μL of 2× kinase solution was added to each well. The plate was incubated at 25°C for 10 minutes. 2 μL of 2× ATP/substrate solution was added to each well. The plate was centrifuged at 1000 rpm for 1 minute. The plate was incubated at 25°C for 60 minutes. 4 μL of ADP-Glo reagent was added to each well. The plate was centrifuged at 1000 rpm for 1 minute. The plate was incubated at 25°C for 40 minutes. 8 μL of detection solution (presumably ADP-Glo detection solution) was added to each well. The plate was centrifuged at 1000 rpm for 1 minute. The plate was incubated at 25°C for 40 minutes. Luminescence signals were measured using a multifunction plate reader.

### 2.4 Thermodynamic solubility

Approximately 1.0 mg of each compound was weighed into three separate 1.5 mL glass vials. To each vial, 1000 µL of assay buffer was added to achieve a final concentration of 1 mg/mL. A PTFE-encapsulated stainless steel stick stirrer was placed in each vial, which was then sealed with a molded PTDE/SIL silicone plug. The sample plate was shaken at 1100 rpm at 25°C for 24 hours. Post-incubation, the samples were filtered, and 10 µL of the samples was mixed with 10 µL of DMSO. The mixture was then added to 980 µL of methanol, then further diluted 10-fold with methanol:water (1:1) for LC-MS/MS analysis.

### 2.5 Metabolic stability study in liver microsomes

Human and mouse liver microsomes in PBS were added to a 96-well incubation plate along with 1 mM NADPH solution and 2 mM UDPGA solution. The reaction was initiated by adding 2 μL of the compound working solution to the plate, carried out at 37°C with shaking at 60 rpm. At 0.5, 15, 30, 45, and 60 minutes, 50 μL samples were transferred to a sample plate containing 200 μL of cold methanol with internal standards (50 nM alprazolam, 50 nM labetalol, and 100 nM ketoprofen). The sample plate was centrifuged at 3,220 g for 40 minutes. Subsequently, 100 μL of the supernatant was transferred to an analysis plate containing water for LC-MS/MS analysis.

### 2.6 Caco-2 permeability assay

Caco-2 cells were cultured in 10% FBS DMEM for 14-21 days before assays. For A-to-B direction, 5 μM compound solution was added to the upper chamber; for B-to-A, to the lower chamber. HBSS was added to the opposite chamber. After 2 hours incubation at 37°C, samples were analyzed by LC-MS/MS. Fluorescein leakage was tested by adding 100 μM fluorescein to the upper chamber and measuring fluorescence after 30 minutes.

### 2.7 *In vivo* pharmacokinetics

Female CD1 mice (6-8 weeks old, 20-30 g) were obtained from SPF Biotechnology Technology Co., Ltd. (Beijing, China). The study was conducted at ICE Bioscience Inc (Beijing, China). Compounds were diluted in a vehicle of 10% DMSO + 15% Solutol HS + 75% (20% SBE-β-CD) for intravenous injection into the mouse tails at a concentration of 10 mg/kg. For oral administration, compounds were diluted in a vehicle of 0.5% MC + 1% Tween-20 with a final dosage of 10 mg/kg. All mice were fasted before dosing. Each route included three animals. Blood samples were collected at 0, 5, 15, 30 minutes; 1, 2, 4, 7, and 24 hours, using EDTA-K2 as the anticoagulant. Samples were centrifuged at 4,000 g, 4°C for 5 minutes, and the plasma were collected and stored at -75°C. The compound concentration in the plasma were analyzed by LC-MS/MS.

### 2.8 Pharmacokinetics and pharmacodynamics in M-NSG mice bearing PSN1 tumor

For *in vivo* PK/PD studies, a PSN1 tumor model was established in female M-NSG (NOD.Cg-*Prkdc^scid^Il2rg^em1Smoc^*) mice, which are severely immunodeficient mice lacking the interleukin 2 receptor gamma chain on a NOD.Cg-*Prkdc^scid^* background, at Shanghai Model Organisms (Shanghai, China). Briefly, 5 × 10^6^ PSN1 cells were inoculated subcutaneously into each M-NSG mouse. Once the tumors reached approximately 500 mm³, compounds were administered orally at various dosages. For PK studies, blood samples were collected at 0, 0.25, 0.5, 1, 2, 6, and 24 hours post-dose. The sera from blood were then obtained and stored at -70°C. For PD studies, tumors were harvested 2 hours after dosing and stored at -70°C.The serum and tumor samples were then transported to PRECEDO (Hefei, Anhui, China) for further PK/PD studies.

For PK studies, serum and tumor samples were processed with the internal standard solution. Serum samples were centrifuged and mixed directly, while tumor samples were homogenized first. Supernatants were prepared for LC-MS/MS analysis. Compound concentrations were determined using LC-MS/MS with tolbutamide as the internal standard, employing a reverse-phase column and ESI on a tandem quadrupole mass spectrometer. Quantitative ranges were 2.00-2000 ng/mL for PR00012 and NMS-P937. Data were processed using AnalystTM 1.7.2 and Excel 2010, with pharmacokinetic parameters calculated using WinNonlin 8.2.

For PD study, tumor tissues (~30 mg) were homogenized in 350 µL Lysis Buffer. After centrifugation, protein concentration in supernatants was determined by BCA assay. Samples were prepared for SDS-PAGE and run on 10% gels. Proteins were transferred to PVDF membranes, blocked, and incubated with anti-Phospho-TCTP (Ser46) Antibody (Cell Signaling Technology, Danvers, MA, USA) (1:1,000) overnight at 4°C. After washing, membranes were incubated with HRP-anti-Rabbit IgG (Cell Signaling Technology, Danvers, MA, USA) (1:3,000) for 1 hour, then treated with ECL reagents for imaging.

### 2.9 M-NSG mice toxicity study

This study was conducted at Shanghai Model Organisms (Shanghai, China). Female M-NSG mice, averaging 25 g in body weight, were randomly divided into 3 groups, each receiving one of the following treatments: 60 mg/kg for NMS-P937, 60 mg/kg and 120 mg/kg for PR00012. Oral gavage was chosen to match the clinical route, with dosing once daily for 6 days. Mice were monitored for an additional 18 days.

### 2.10 Sprague-Dawley Rat toxicity model

Rat toxicity was studied at JOINN Laboratories (Suzhou, Jiangsu, China). Sprague-Dawley rats were randomly divided into 2 groups, each receiving 15 mg/kg at 1.5 mg/mL, with 3 female animals per group. Group 1 received PR00012, and Group 2 received NMS-P937. Oral gavage was chosen to match the clinical route, with dosing once daily for two weeks at 15 mg/kg. Using 2 mL sterile syringes and gavage tubes, animals were dosed based on their most recent body weight, with volumes rounded to one decimal place. Test compounds were stirred continuously for at least 15 minutes before and during administration. The rats were dosed once a day for 14 days.

### 2.11 Cell line-derived tumor xenografts studies

These studies were performed at Yikon Medical (Beijing, China). Cancer cell line HCT 116 or BxPC-3 were cultured in 10% FBS RPMI-1640 medium plus penicillin, streptomycin, and glutamine at 37°C and 5% CO2 until reaching confluency. Cells were then prepared for inoculation. 5×10⁷ cells/mL cells in 100 µL were inoculated subcutaneously into the right flank of each female BALB/c nude mouse for HCT 116 cell line or female NOD SCID mouse for BxPC-3 cell line. When tumors reached 100-150 mm³, mice were divided into 4-5 groups (6-7 mice/group). Different groups were dosed with Vehicle, PR00012 or NMS-P937 at various concentrations. Mice were dosed at least 10 days and were monitored for 30 days.

### 2.12 Tumor growth inhibition

TGI was calculated as follows. TGI(%)=100×(1−ΔT/ΔC), ΔT represents the volume change for treated tumors and ΔC represents the volume change for control tumors.

### 2.13 Statistical analysis

All statistical analyses were conducted using Prism 10.1.2 (GraphPad). Extra sum-of-squares F test was used. Data was considered statistically significant at p < 0.05.

## 3 Result

### 3.1 Chemistry

**Figure 1:**
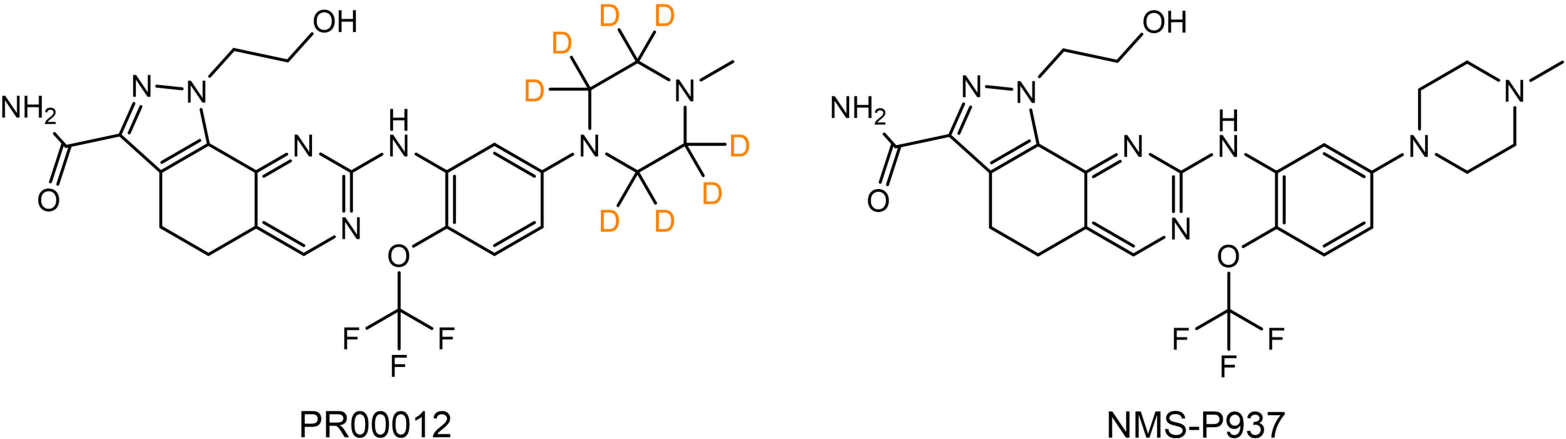
Chemical structures of PR00012 and NMS-P937. Lewis structures of PR00012 and NMS-P937 are shown. All hydrogen atoms on piperazine moiety of NMS-P937 are replaced by deuterium to form the molecule PR00012. D indicates deuterium.

The molecule of PR00012 was generated by replacing all the hydrogen atoms with deuterium on piperazine of the molecule NMS-P937 (**Figure 1**).

### 3.2 Similar cancer cell growth inhibition and kinase inhibition by PR00012 and NMS-P937 compounds

**Figure 2:**
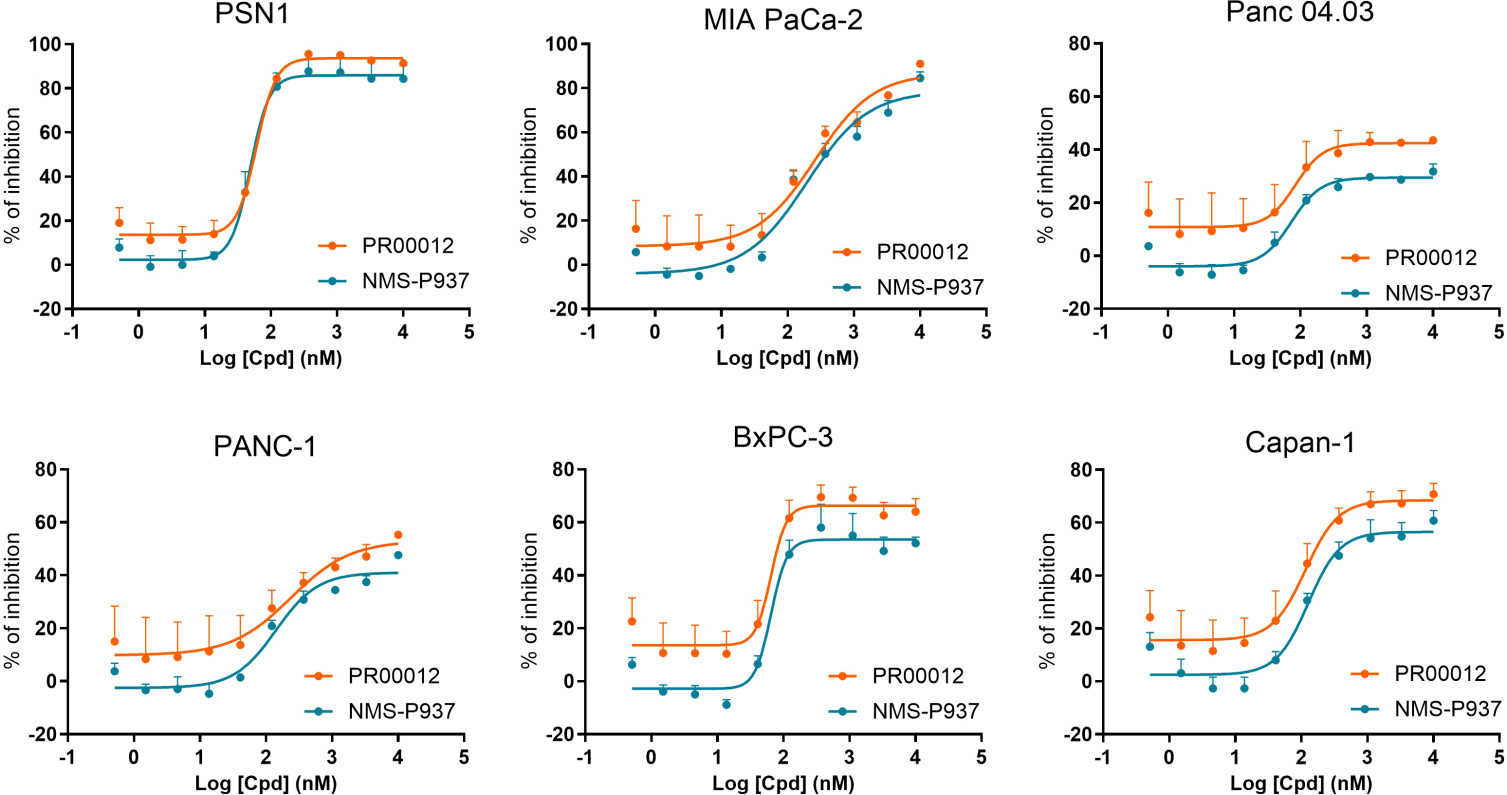
Inhibitory effects of PR00012 and NMS-P937 on pancreatic cancer cell lines. Cell lines were plated in 96-well plates and cultured overnight. Test compounds were then added at various concentrations in triplicates and incubated for 72 hours. Cell viability was finally assessed. The data demonstrated the dose-dependent inhibitory effects of the compounds.

We compared the inhibitory function of PR00012 and its non-deuterated form, NMS-P937, against six human pancreatic cancer cell lines. Cell lines were plated in 96-well plates and cultured overnight. Test compounds were then added at various concentrations in triplicates and incubated for 72 hours. Cell viability was finally assessed. The data demonstrated the dose-dependent inhibitory effects of the compounds. However, there was no significant difference between PR00012 and NMS-P937 (**Figure 2**). Also summarized in **Table 1**, PR00012 displayed similar or slightly higher IC_50_ value compared to NMS-P937, with no significant differences.

**Table 1:** IC_50_ values of PR00012 and NMS-P937 against human pancreatic cancer cell lines.

Cell line	Compound	Top	Bottom	IC_50_ (nM)
PSN1	PR00012	93.7	13.7	60.7
	NMS-P937	85.9	2.3	48.9
MIA PaCa-2	PR00012	86.8	8.4	261.9
	NMS-P937	78.7	-4.1	195.5
Panc04.03	PR00012	42.5	10.8	83.8
	NMS-P937	29.5	-4	73
PANC-1	PR00012	53.1	9.8	236.7
	NMS-P937	41.1	-2.5	141.6
BxPC-3	PR00012	66.3	13.6	65.1
	NMS-P937	53.5	-2.8	64.9
Capan-1	PR00012	68.5	15.6	115.2
	NMS-P937	56.5	2.5	126.9

**Figure 3:**
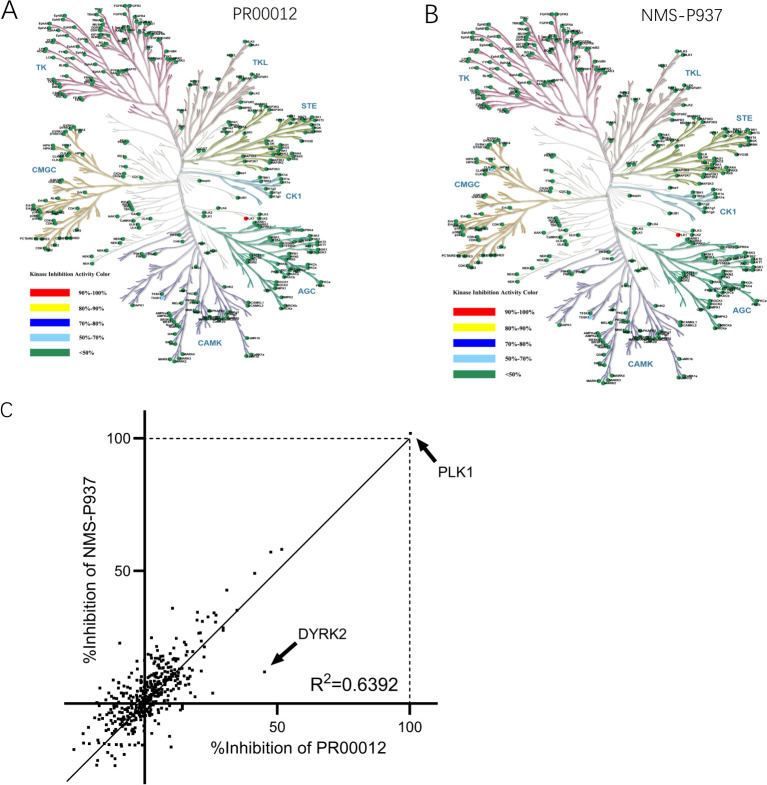
Kinase inhibition activities of PR00012 and NMS-P937. HTFR high-throughput assays were used to screen 416 kinases against PR00012 and NMS-P937 at 0.1 μM. **(A)** Kinase inhibition tree for PR00012. **(B)** Kinase inhibition tree for NMS-P937. **(C)** Scatter plot comparing kinase inhibition by PR00012 and NMS-P937.

Next, we investigated the kinase inhibition ability of PR00012 and NMS-P937 against 416 kinases at the concentration of 0.1 μM. As shown in the kinase trees, the deuterated PR00012 and NMS-P937 exhibited similar kinase activities (**Figures 3A, B**). The correlation in the inhibitory percentage between PR00012 and its non-deuterated counterpart, NMS-P937, was substantial (**Figure 3C**), confirming their similarities in receptor binding affinity. As depicted in **Figure 3C**, the correlation between these two compounds was robust, with an R² value of 0.6392, underscoring their highly similar pharmacological profiles. There are only one notable outlier, which is tyrosine-phosphorylation-regulated kinase 2 (DYRK2) (**Figure 3C**).

We further performed a dose-dependent assay for those kinases inhibited for more than 40% by PR00012 at 0.1 μM to determine their IC_50_ value (**Table 2**). For the 15 kinases tested, PR00012 had a similar IC_50_ value to NMS-P937 among 14 kinases. However, the kinase activity of DYRK2 was significantly inhibited by PR00012, but not NMS-P937. The IC_50_ of NMS-P937 for DYRK2 was about 14-fold higher than PR00012. The function of DYRK2 in tumorigenesis is not clear, it may function either as a promoter or inhibitor of cancer, depending on the organ context (20). However, DYRK2 Inhibitor has been developed for the treatment of prostate cancer (21). Furthermore, DYRK2 was targeted by a small molecule LDN192960 for the treatment of for triple-negative breast cancer and multiple myeloma (22). In addition, high expression of DYRK2 led to lower survival probability in pancreatic cancer, breast cancer, and renal cancer, among others (proteinatlas.org).

**Table 2:** The IC_50_ values of PR00012 and NMS-P937 against 15 kinases.

Assay	IC_50_ (nM)	
	PR00012	NMS-P937
PLK1	0.13	0.22
PLK2	>10000	>10000
DYRK2	81.4	1154.0
CK1δ	51.8	59.3
FLT3 D835Y	184.4	201
CLK2	24.8	37.0
CDK9/CycT1	720.6	648.1
RON	904.7	1089.0
CK1ϵ	156.0	156.4
TSSK1	53.2	77.8
DRAK1	48.7	44.6
DYRK3	112.1	114.2
CLK1	196.5	234.5
CK1α	181.8	184.2
CLK4	231.2	278.1

### 3.3 Metabolic stability after the compound NMS-P397 are deuterated

The metabolic stability was assessed by assays using human and mouse liver microsomes. PR00012 exhibits a slightly longer T_1/2_ (half-life) and a slightly slower clearance rate in both human and mouse liver microsomes than NMS-P937 (**Table 3**). In human liver microsomes, the T_1/2_ was 90 minutes and 86 minutes for PR00012 and NMS-P937, respectively (**Table 3**). The T_1/2_ in mouse liver microsomes was 76 minutes and 66 minutes for PR00012 and NMS-P937, respectively (**Table 3**). The intrinsic clearance (CLint) in human liver microsomes was 15 and 16 μL/min/mg protein for PR00012 and NMS-P937, respectively (**Table 3**). In mouse liver microsomes, the CLint was 18 and 21 μL/min/mg protein for PR00012 and NMS-P937, respectively (**Table 3**).

**Table 3:** *In vitro* metabolic stability in human and mouse liver microsomes.

Compound	Species	T1/2 (min)	CLint
			(µL/min/mg protein)
PR00012	Human	89.9	15.4
NMS-P937		86.4	16.1
PR00012	Mouse	76.3	18.2
NMS-P937		66.4	20.9

​The Caco-2 permeability assay is critical for evaluating the intestinal absorption of compounds as well as the compound permeability in tissues. AS shown in **Table 4**, by comparing Propranolol, Digoxin, and Prazosin to our test compounds, PR00012 and NMS-P937 are characterized as candidate drugs with medium permeability. Their efflux ratios are similar, and the recoveries showed no significant difference.

**Table 4:** Caco-2 permeability data.

Compound	Papp _A-B_	Papp _B-A_	Efflux	Recovery	Recovery
	(1E-6 cm/s)	(1E-6 cm/s)	Ratio	A-B%	B-A%
Propraolol	22.3	14.7	0.7	61.6	84.9
Digoxin	0.9	26.1	27.8	78.5	101.1
Prazosin	9	29.6	3.3	71.8	80.9
PR00012	5.2	34.7	6.7	79.7	89.7
NMS-P937	6.1	34.2	5.6	71.6	93.3

As previously described, the lower energy state of the carbon-deuterium (C-D) bond hinders the ability of drug transporters and enzymes to metabolize the compound efficiently, thereby reducing its rate of metabolism (3). Deuterated drugs, which incorporate deuterium in place of hydrogen, take advantage of this kinetic isotope effect. Deuterium, being twice as heavy as hydrogen, forms stronger C-D bonds compared to C-H bonds. This subtle change can significantly impact the pharmacokinetics of the drug, including its absorption, distribution, metabolism, and excretion (ADME) properties (7).

Since our compound is intended to treat cancer patients such as those with pancreatic cancer, it is important to evaluate the PK and PD properties of PR00012 and NMS-P937 in tumor-bearing mice.

The PSN-1 cell line, derived from a human pancreatic adenocarcinoma, is characterized by specific genetic mutations that are significant for cancer research. A critical mutation present in the PSN-1 cell line is a point mutation in the *KRAS* gene. This mutation involves a substitution from GGT to CGT at codon 12 of the gene, which is presented at *KRAS* G12R, leading to a 3 to 6-fold amplification of *KRAS*mRNA *(* 23). In addition, PSN-1 cell lines were previously found to have 50-fold amplification of *c-Myc* mRNA (23), while the aberrant high expression of *c-Myc* has been associated with more aggressive cancer phenotypes.

**Figure 4:**
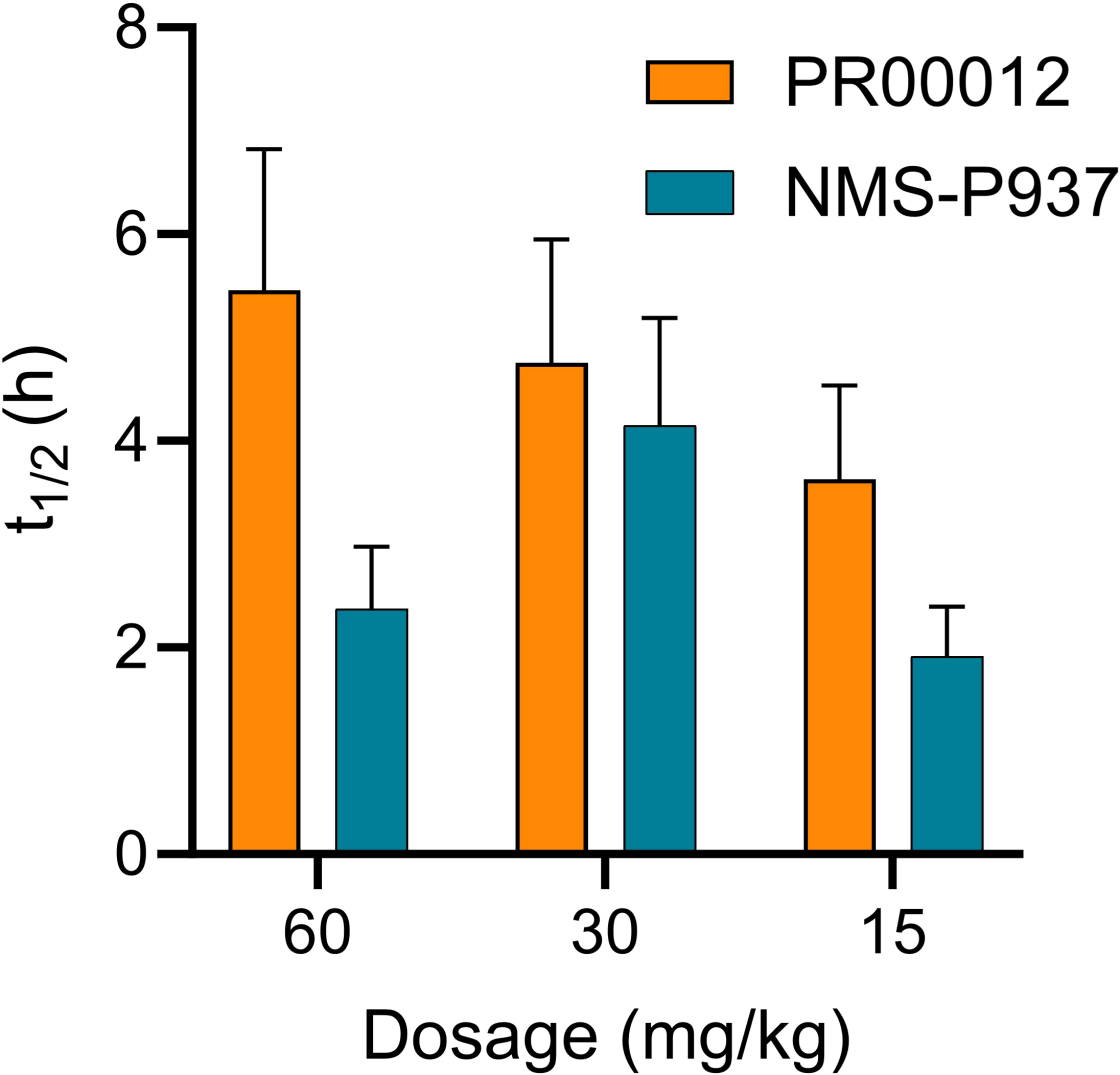
Pharmacokinetics of PR00012 and NMS-P937 in PSN1 tumor CDX model using M-NSG mice. The tumor-bearing M-NSG mice were dosed with PR00012 or NMS-P937 at 60 mg/kg, 30 mg/kg, or 15 mg/kg. Blood samples were collected at seven time points. Compound concentration in the blood were determined and theT1/2 was calculated.

Here we established PSN-1 CDX model in M-NSG mice. M-NSG mice are a modified version of the NOD scid gamma (NSG) mouse strain. These mice lack mature T cells, B cells, and natural killer (NK) cells. M-NSG mice further enhance this immunodeficiency, enabling even higher efficiency in the engraftment of human cells. The PNS-1 tumor-bearing mice were dosed with PR00012 and NMS-P937 at 60 mg/kg, 30 mg/kg or 15 mg/kg, and the blood samples were collected at seven time points. The compound concentration in the blood were determined and theT_1/2_ was calculated. As shown in **Figure 4**, the T_1/2_ of PR00012 was consistently higher across all three concentrations than NMS-P937. When mice were dosed at 60 mg/kg, the T_1/2_ were 5.5 h and 2.4 h for PR00012 and NMS-P937, respectively (**Figure 4**). Similar data were observed after mice were dosed at 30 mg/kg and 15 mg/kg.

Thus, PR00012, a deuterated NMS-P937 compound, demonstrates slightly high T_1/2_ in both *in vitro* liver microsomes and *in vivo* tumor-bearing mice.

### 3.4 Enhanced inhibition of PLK1-induced phospho- translationally controlled tumor protein

To thoroughly evaluate pharmacodynamic effect of PLK1 inhibition *in vivo*, tumors from the PSN-1 xenograft tumor model in M-NSG mice were collected at 2 and 6 hours following a single oral dose of the compounds at varying concentrations (60, 30, and 15 mg/kg). Western blot analysis was performed to assess the levels of p-TCTP, a direct substrate of PLK1. Phosphorylation of TCTP has been demonstrated as a marker for PLK1 activity *in vivo* (15, 24).

**Figure 5:**
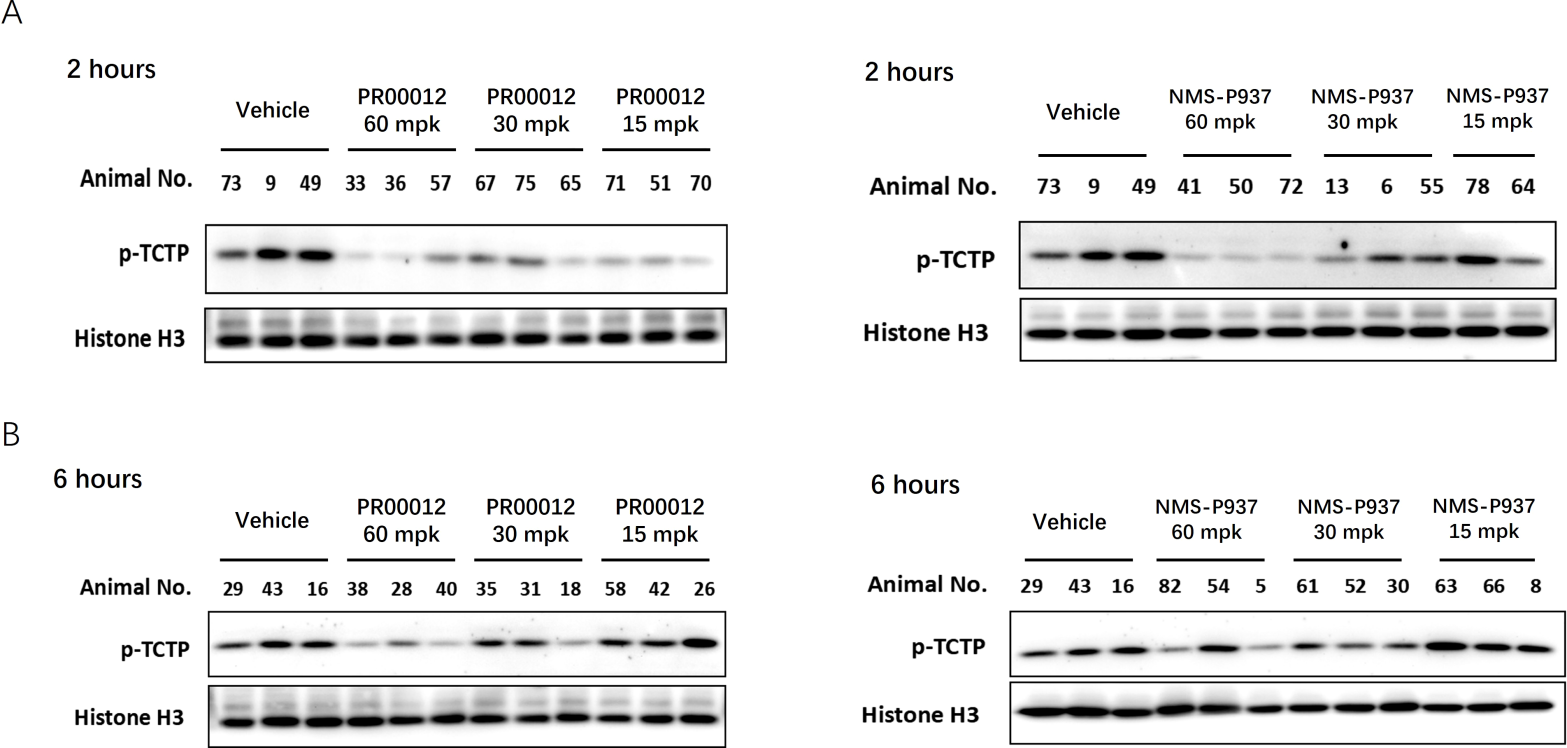
Inhibition of p-TCTP in tumors following treatment with PR00012 and NMS-P937. PSN-1 xenograft tumor models were established in M-NSG mice. Mice subsequently received single oral doses of PR00012 or NMS-P937 at 60, 30, or 15 mg/kg. Mice were sacrificed at designated time points. Phospho-Ser46 TCTP expression in tumors was assessed using Western blotting. **(A)** Tumors obtained 2 hours post-dosing. **(B)** Tumors obtained 6 hours post-dosing.

After 2-hour *in vivo* treatment, both PR00012 and NMS-P937 exhibited reduced p-TCTP signal, indicating a decrease in PLK1 activity (**Figure 5A**). Notably, PR00012 treatment significantly reduced TCTP phosphorylation compared to NMS-P937 treated group, particularly at the lower doses. This suggests that PR00012 has a stronger PLK1 inhibition capability. Six hours after administration of the PLK1 inhibitors, p-TCTP levels from both drugs began to return to baseline (**Figure 5B**), demonstrating a time-dependent PLK1 inhibition effect.

In summary, both PR00012 and NMS-P937 effectively inhibit PLK1 activity *in vivo*, with PR00012 demonstrating a stronger inhibition of PLK1 in PSN-1 xenograft tumors. These findings suggest that PR00012 may offer enhanced therapeutic efficacy in targeting PLK1-related pathways for tumor growth suppression.

### 3.5 Reduced *in vivo* toxicity by PR00012

**Figure 6:**
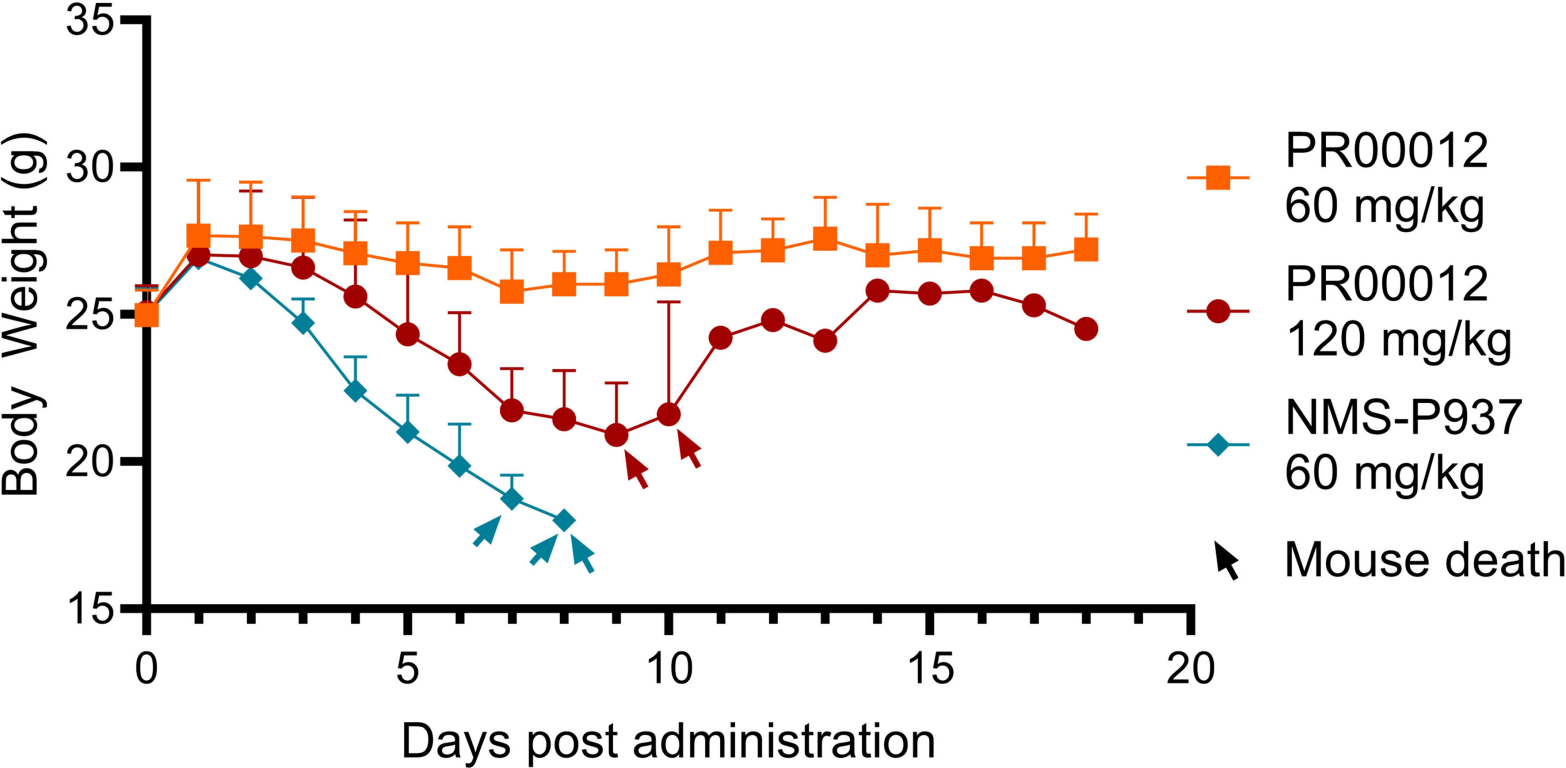
Body weight and survival of M-SNG mice treated with PR00012 and NMS-P937. PR00012 or NMS-P937 were orally administered to M-NSG mice for the first six days, followed by a 12-day monitoring period. Each arrowhead represents the death of one mouse.

We initially aimed to investigate the *in vivo* toxicity and efficacy using M-NSG mice. Mice were received oral administration of both compounds for the first six days, followed by an observation period until the 18th day. In the group treated with NMS-P937 at a dose of 60 mg/kg, all three mice significantly lost body weight, and eventually died before day 9 (**Figure 6**). Conversely, in the group treated with PR00012 at a double dosage of NMS-P937 (120 mg/kg), only two out of three mice died before day 11. Interestingly, when PR00012 was administered at 60 mg/kg, the same dosage of NMS-P937, all mice survived throughout the observation period without significant loss of body weight.

**Figure 7:**
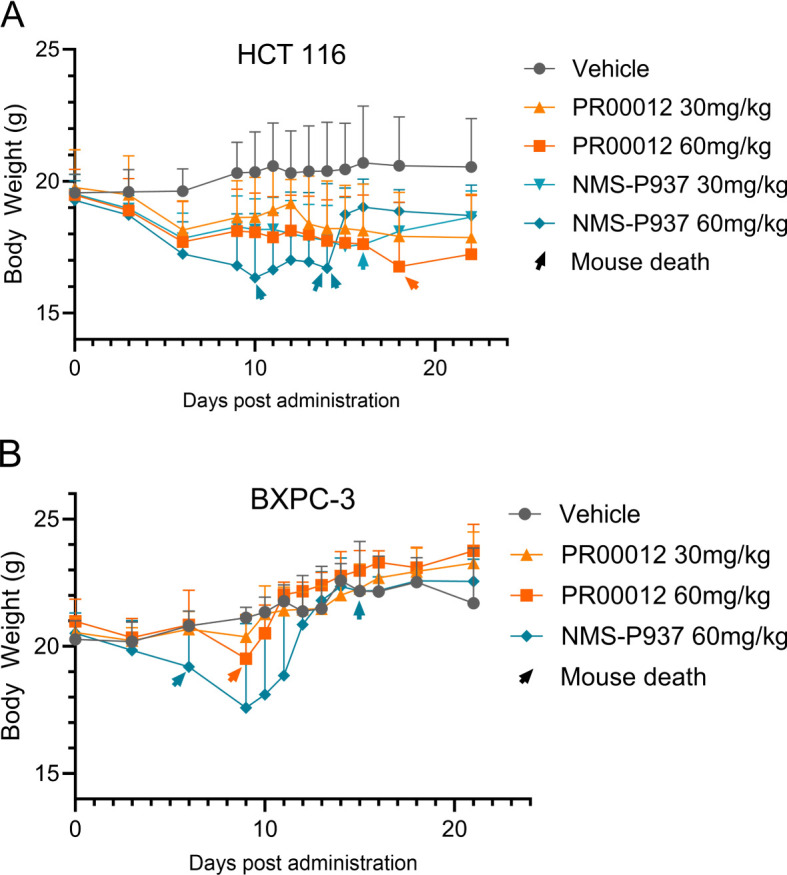
Body weight and survival in CDX models using BALB/c nude and NOD SCID mice. **(A)** HCT 116 CDX model: 5×10^6^ HCT 116 cells were inoculated subcutaneously into the right flank of BALB/c nude mouse. When tumors reached 100-150 mm³, mice were divided into 5 groups (8 mice/group) and dosed with vehicle, PR00012 or NMS-P937 at different concentration. Treatment lasted 16 days (10 days for NMS-P937 60 mg/kg group) with 22-day monitoring. Each arrowhead represents the death of one mouse. 1/8 mouse died after dosing with NMS-P937 at 30 mg/kg, 3/8 mice died after dosing with NMS-P937 at 60 mg/kg, 1/8 mouse died after dosing with PR00012 at 60 mg/kg. No death at other concentrations. **(B)** BxPC-3 CDX model: 5 ×10^6^ BxPC-3 cells were inoculated subcutaneously into the right flank of NOD SCID mice. When tumors reached 100-150 mm³, mice were divided into 4 groups (6 mice/group) and dosed with vehicle, PR00012 or NMS-P937 at different concentration. Treatment lasted 10 days. Each arrowhead represents the death of one mouse. 2/6 mice died after dosing NMP-P937 at 60 mg/kg while only 1/6 mouse died after dosing PR00012 at 60 mg/kg. No death reported on other concentrations.

In addition, in our CDX efficacy models, we observed higher mortality rates in NMS-P937-treated mice compared to those treated with deuterated PR00012, regardless of whether the mice were from BALB/c nude or NOD SCID background (**Figures 7A, B**). In the HCT 116 CDX studies, there were three mice died in the NMS-P937 (60 mg/kg)-treated group while only one mouse died in the PR00012 (60 mg/kg)-treated group. No mice died in the PR00012 (30 mg/kg) group, whereas one mouse died when dosed with NMS-P937 at 30 mg/kg (**Figure 7A**). In the BxPC-3 CDX studies, two mice died in the NMS-P937 dosed group while only one mouse died in PR00012 dosed group (**Figure 7B**). Furthermore, when mice dosed at 60 mg/kg, the body weights were significantly lower in the NMS-P937-dosed group (**Figures 7A, B**).

We extended our toxicity studies to include a rat model. Initially, we evaluated the response of different dosages of PR00012 in Sprague-Dawley rats. At 10 mg/kg, the rats were still tolerable (data not shown).

**Figure 8:**
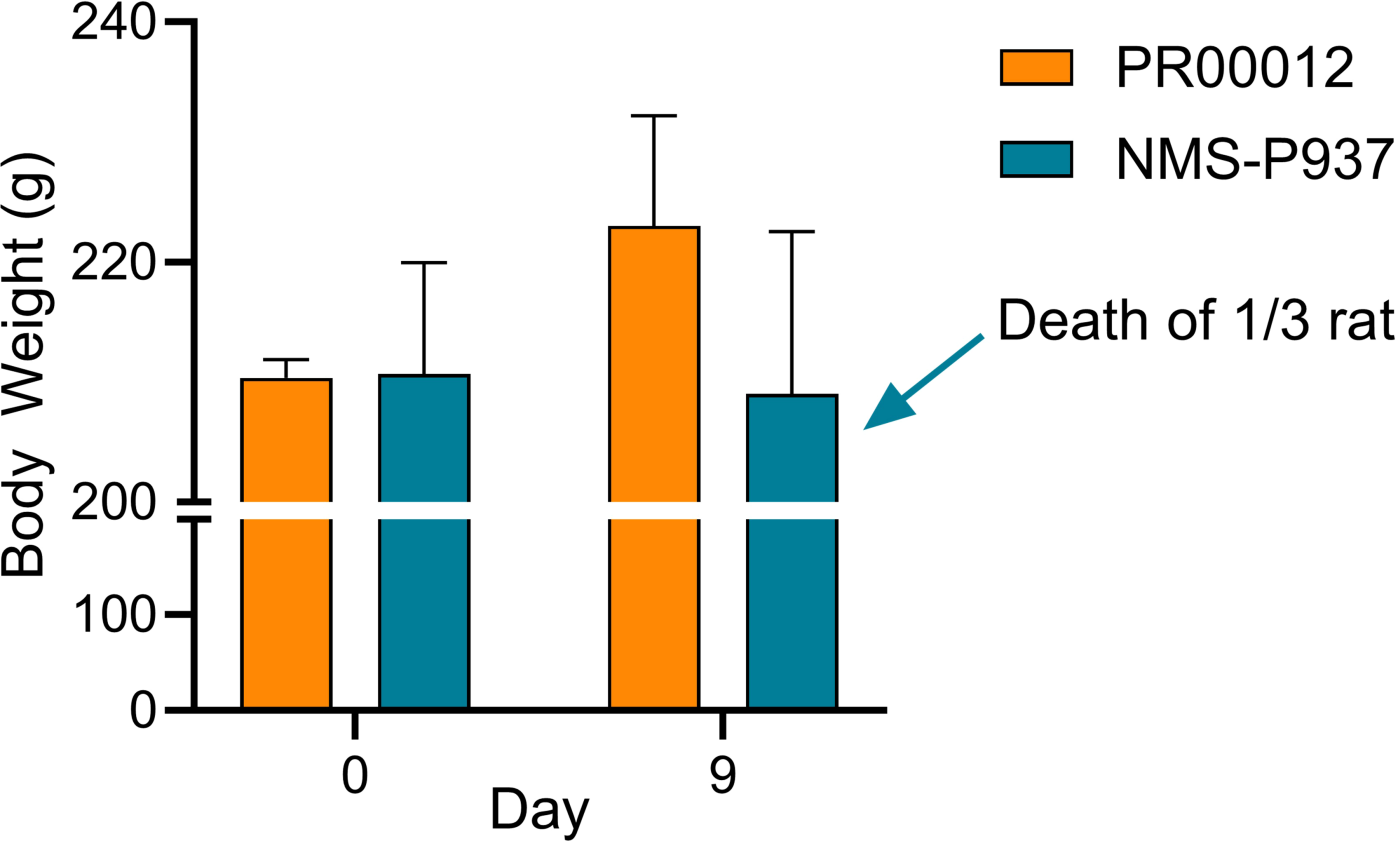
Body weight and survival of Sprague-Dawley rats treated with PR00012 and NMS-P937. Rats were dosed with PR00012 or NMS-P937 at 15 mg/kg every day for 14 days. All three rats (3/3) survived at day 14 with PR00012 treatment. For NMS-P937, one rat died at day 9, the remaining two rats (2/3) survived at day 14.

Subsequently, we conducted a 14-day repeated administration toxicity study. Rats were orally dosed with PR00012 or NMS-P937 at 15 mg/kg once a day. After 9-day dosing, all three rats treated with PR00012 survived, while 1 out of 3 rats treated with NMS-P937 died (**Figure 8**). The rat that died exhibited severe symptoms, including arching back, watery diarrhea, decreased spontaneous activity, pale ears, perianal soiling, ruffled fur, yellowing of fur around the groin, urethra, and vaginal area, and weight loss (data not shown). The body weight of NMS-P937-treated rats were also lower than PR00012-treated group (**Figure 8**). Upon completion of the 14-day dosing, all PR00012-treated rats remained alive.

These findings suggest that PR00012 possesses a more favorable safety profile compared to NMS-P937, particularly when administered at the same dosage level. The improved survival rates and minimal weight impact highlight PR00012’s potential as a safer therapeutic option.

### 3.6 CDX studies demonstrated slightly better *in vivo* tumor growth inhibition by PR00012

CDX models are essential in preclinical oncology research. They allow for the evaluation of anti-cancer therapies through the implantation of *in vitro* cultured human cell lines into immunocompromised mice, creating a tumor-bearing system.​ These models facilitate the assessment of drug efficacy, pharmacokinetics, and the underlying mechanisms of action in an *in vivo* environment. CDX models are valued for their reproducibility and ability to mimic human tumor characteristics, providing a controlled environment to test various therapeutic agents and analyze tumor growth kinetics. This makes them a practical choice for preclinical evaluation.

**Figure 9:**
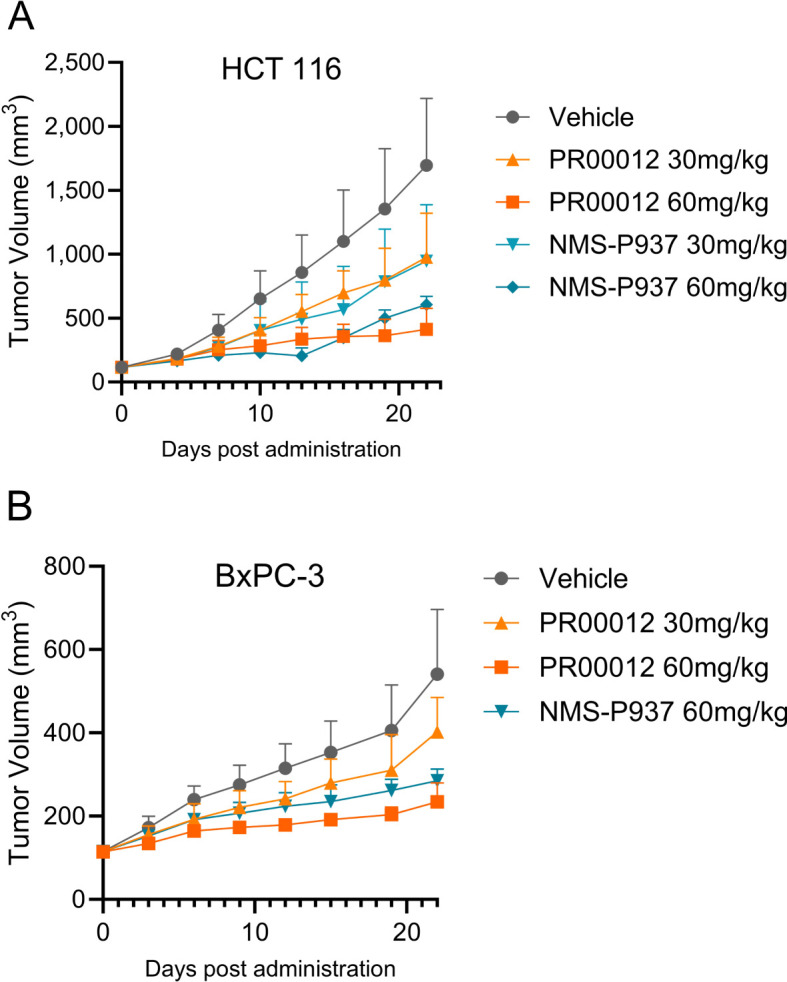
Tumor growth inhibition by the treatment of PR00012 and NMS-P937 in mouse CDX models. **(A)** HCT 116 CDX model: Experimental setup as described in **Figure 8A**. Mice were dosed for 16 days (10 days for NMS-P937 60 mg/kg group). Tumor volume was measured during the 22-day period. **(B)** BxPC-3 CDX model: Experimental setup as described in **Figure 8B**. Mice were dosed for 10 days. Tumor volume was measured during the 22-day period. Data were representative of two similar experiments.

We first established CDX model in BALB/c nude mice using HCT116 cells, a human colon cancer cell line. Our compounds were tested after the models were established. Both PR00012 and NMS-P937 inhibited the growth of tumors in a dose-dependent manner. The TGI of PR00012 (60 mg/kg)-treated group was slightly better than that of NMS-P937-treated group (**Figure 9A**). In addition, more mice died in the NMS-P937-treated groups, and there was significant loss of body weight in the NMS-P937 (60 mg/kg)-treated group (**Figure 7A**).

For pancreatic cancer cell lines, we have established two CDX models: one with deletion of the *SMAD4* gene and the other from a *KRAS* mutation cell line.

#### 3.6.1 BxPC-3 model

The BxPC-3 cell line is characterized by specific genetic alterations typical of pancreatic cancer. Notably, it lacks the KRAS mutation and features a homozygous deletion of the SMAD4 gene (25), making it a valuable resource for studying the genetic landscape of pancreatic cancer.

After establishing the CDX model in NOD SCID mice, mice were grouped and dosed with different dosage of PR00012 and NMS-P937. PR00012-treated mice displayed tumor growth inhibition in a dose-dependent manner (**Figure 9B**). At 60 mg/kg PR00012-treated mice showed slightly more tumor growth inhibition than NMS-P937-treated group (**Figure 9B**). Furthermore, NMS-P937-dosed mice exhibited dramatically reduced body weight, while PR00012-dosed mice did not. More mice died from NMS-P937 treatment compared to PR00012 (**Figure 7B**).

#### 3.6.2 PSN-1 model

As discussed earlier, the PSN-1 pancreatic cell line harbors a KRAS G12R mutation as well as high amplification of c-Myc (23). Aberrant high expression of c-Myc has been associated with more aggressive cancer phenotypes. The PSN-1 CDX model was established in M-NSG mice, a modified version of NSG mouse strain. However, we have found the M-NSG mice were more sensitive to our compound treatment as showed decreased body weight and death (**Figure 6**). Mice started to die at day 7 after NMS-P937 treatment. Due to this sensitivity, we compared the tumor growth inhibition in the PSN-1 CDX model in M-NSG mice after the mice were treated with two compounds for 4 days. Both compounds inhibited the PNS-1 tumor growth in dose-dependent manners (**Table 5**). For comparison, PR00012 inhibited the growth of tumor slightly better than NMS-P937 in all three doses (**Table 5**).

**Table 5:** TGI Comparison after 4-day dosing in the PSN-1 CDX model.

Compound	Dose	Tumor volume (mm^3^)		TGI (%)	P value
		Day 0	Day 4		
Vehicle		119.46 ± 1.77	215.39 ± 8.25	N/A	N/A
PR00012	60 mg/kg	119.48 ± 1.72	127.93 ± 1.60	91.2	<0.01
NMS-P937		119.42 ± 2.14	137.49 ± 5.16	81.2	<0.01
PR00012	30 mg/kg	119.48 ± 1.95	143.49 ± 5.64	75.0	<0.01
NMS-P937		119.38 ± 2.09	149.94 ± 4.21	68.1	<0.01
PR00012	15 mg/kg	119.44 ± 1.78	162.12 ± 4.11	55.5	<0.01
NMS-P937		119.66 ± 1.67	166.43 ± 5.79	51.3	<0.01

In summary, across three different CDX models, PR00012 consistently demonstrated slightly superior tumor growth inhibition compared to NMS-P937.

## 4 Discussion

In this study, we generated PR00012 by replacing all the hydrogens with deuterium on piperazine of the molecule NMS-P937. Most of the characteristics of deuterated-PR00012 are similar to NMS-P937, with some notable improvements. PR00012 demonstrates slightly higher T_1/2_ *in vivo* than non-deuterated NMS-P937. The inhibition of p-PCTP by PLK1 in tumors was stronger by PR00012 treatment in tumor-bearing mice. PR00012 exhibits significantly reduced toxicity, as evidenced by lower mortality rates in mice with three different genetic backgrounds and in Sprague-Dawley rats. These findings reinforce the potential of PR00012 as a safer alternative for therapeutic applications, offering improved tolerability and a more favorable therapeutic profile compared to NMS-P937.

Deuterated drugs are recognized for their enhanced pharmacokinetic and pharmacodynamic properties, including increased stability *in vivo* due to reduced CYP450 metabolism, decreased toxicity and side effects, improved half-life, as well as reduced off-target effects (26, 27). PR00012 aligns with these beneficial characteristics, showcasing its potential as a superior therapeutic agent. For example, deutetrabenazine, a deuterated version of tetrabenazine, exhibits significantly improved metabolic stability and a longer half-life due to decreased CYP450 metabolism, leading to a better therapeutic profile and reduced side effects compared to its non-deuterated counterpart (28, 29). These examples illustrate the potential advantages of deuterated drugs, supporting the observation that PR00012 demonstrates a slightly reduced clearance rate. This lower clearance rate, consistent with reduced CYP450 metabolism, aligns with findings in other deuterated compounds (30).

The *in vivo* half-life of PR00012 in M-NSG mice is longer than that of NMS-P937, comparable to the extended half-life observed in deuterated drugs like deuterated L-dopa, which shows improved pharmacokinetics and a longer half-life in patients with Parkinson’s disease (28). This extended half-life of PR00012 suggests that it remains more stable within the biological system, maintaining therapeutic levels for an extended duration and potentially requiring less frequent dosing.

Moreover, the pharmacodynamics of PR00012, as measured by PLK1-induced phospho-TCTP levels, are slightly superior to those of NMS-P937. This suggests that PR00012 not only maintains the desired biological activity but also offers improved stability and safety profiles. This is similar to the pharmacodynamic enhancements observed with deuterated drugs such as deuterated tamoxifen, which retains its efficacy while offering improved pharmacodynamics properties (31).

The enhanced *in vivo* efficacy of PR00012 could be partially attributed to its inhibition of DYRK2. From **Table 2** and **Figure 3**, most of the IC_50_ values for the kinases are highly similar between PR00012 and NMS-P937, except for DYRK2. DYRK2 influences the G1/S and G2/M cell cycle transitions by phosphorylating p53, which activates p21, leading to G1 phase arrest for DNA repair. It also regulates proteasome activity during the G2/M transition to ensure proper mitotic entry and progression, maintaining genomic integrity and preventing uncontrolled cell proliferation (32). DYRK2 has a lower IC_50_ for PR00012, demonstrating stronger inhibition, different from most other kinases. DYRK2 directs cells into the apoptosis cascade, terminating the cell cycle and reducing phosphorylated NPM concentration (33). This suggests that PR00012 might perform variably across different tissues, similar to how DYRK2’s functions differ in various tissue types. For instance, DYRK2’s regulatory roles in cell survival, differentiation, and apoptosis are tissue-dependent, affecting breast cancer differently than other cancers (34). While DYRK2 does have tumor suppressor-like functions (such as p53 phosphorylation), it has pro-cancer functions that appear to be dominant in certain cancer types. This dual nature of DYRK2 (both pro- and anti-cancer effects) is not unusual for kinases. DYRK2 Inhibitor thus has been developed for the treatment of prostate cancer, triple-negative breast cancer and multiple myeloma (21, 22).

​Additionally, TCTP plays a significant role in tumor development primarily by promoting cell proliferation, inhibiting cell death, and enhancing cell migration.​ Its expression levels correlate with various cancer types, and it has been identified as a potential therapeutic target due to its influence on cancer progression. The better *in vivo* tumor growth inhibition observed with PR00012 could be due to its more effective inhibition of p-TCTP compared to NMS-P937.

In terms of safety, PR00012 demonstrates a more favorable profile compared to NMS-P937. This is evident in all three mice models (**Figures 6**, **7**), where mice treated with PR00012 show less weight loss and higher survival rates than those treated with NMS-P937 at the same dosage. In the M-NSG mice model, even at twice the dosage of NMS-P937, PR00012 maintains better survival rates (**Figure 6**). In rat model, one rat died in the group treated with NMS-P937 while no rat died in PR00012-dosed group. This enhanced safety profile is consistent with the improved safety observed in other deuterated drugs, where the substitution of hydrogen with deuterium leads to reduced toxicity and side effects (35).

In conclusion, PR00012 exemplifies the favorable attributes of deuterated drugs, with its slightly reduced clearance rate, extended half-life, better safety profile and slightly better tumor growth inhibition, making it a promising candidate for further development. These characteristics underscore its potential to deliver more effective and safer therapeutic interventions, particularly in comparison to NMS-P937.

## Data Availability

The original contributions presented in the study are included in the article/supplementary material. Further inquiries can be directed to the corresponding authors.
